# Commercial Wearables for the Management of People with Autism Spectrum Disorder: A Review

**DOI:** 10.3390/bios14110556

**Published:** 2024-11-15

**Authors:** Jonathan Hernández-Capistrán, Giner Alor-Hernández, Humberto Marín-Vega, Maritza Bustos-López, Laura Nely Sanchez-Morales, Jose Luis Sanchez-Cervantes

**Affiliations:** 1 Tecnológico Nacional de México/I.T. Orizaba, Av. Oriente 9, 852. Col. Emiliano Zapata, Orizaba C.P. 94320, Veracruz, Mexico; jonathan.hc@orizaba.tecnm.mx (J.H.-C.); humberto.mv@orizaba.tecnm.mx (H.M.-V.); maritza.bl@orizaba.tecnm.mx (M.B.-L.); jose.sc@orizaba.tecnm.mx (J.L.S.-C.); 2 CONAHCYT—Tecnológico Nacional de México/I.T. Orizaba, Av. Oriente 9, 852. Col. Emiliano Zapata, Orizaba C.P. 94320, Veracruz, Mexico; laura.sanchez@conahcyt.mx

**Keywords:** autism spectrum disorder, sensors, wearables, physiological

## Abstract

Autism Spectrum Disorder (ASD) necessitates comprehensive management, addressing complex challenges in social communication, behavioral regulation, and sensory processing, for which wearable technologies offer valuable tools to monitor and support interventions. Therefore, this review explores recent advancements in wearable technology, categorizing devices based on executive function, psychomotor skills, and the behavioral/emotional/sensory domain, highlighting their potential to improve ongoing management and intervention. To ensure rigor and comprehensiveness, the review employs a PRISMA-based methodology. Specifically, literature searches were conducted across diverse databases, focusing on studies published between 2014 and 2024, to identify the most commonly used wearables in ASD research. Notably, 55.45% of the 110 devices analyzed had an undefined FDA status, 23.6% received 510(k) clearance, and only a small percentage were classified as FDA Breakthrough Devices or in the submission process. Additionally, approximately 50% of the devices utilized sensors like ECG, EEG, PPG, and EMG, highlighting their widespread use in real-time physiological monitoring. Our work comprehensively analyzes a wide array of wearable technologies, including emerging and advanced. While these technologies have the potential to transform ASD management through real-time data collection and personalized interventions, improved clinical validation and user-centered design are essential for maximizing their effectiveness and user acceptance.

## 1. Introduction

Autism Spectrum Disorder (ASD) is a neurodevelopmental condition marked by ongoing difficulties in social communication, restricted interests, and repetitive behaviors, with varying severity and support needs across individuals [1]. The term ASD has been widely adopted in the modern scientific literature due to its ability to describe the wide clinical variability of the disorder, allowing for a more inclusive approach than the previously used term autism [2]. This shift is aligned with the DSM-5 guidelines, which integrate several conditions previously considered separate, such as Asperger’s Syndrome, under one category to reflect the diversity within the spectrum [3]. The use of ASD favors a better understanding by health professionals and the public, promoting a less stigmatizing and more comprehensive approach [4]. In addition, it facilitates more accurate diagnoses and implementations of personalized interventions, essential to addressing the specific needs of each patient [5]. People with ASD often face challenges in social interactions and display unusual behavioral patterns, such as intense focus on details and atypical sensory responses [6]. This heterogeneity in symptoms complicates its characterization and the consistency of research findings [7]. As ASD prevalence has risen, there is an increased demand for effective diagnostic and management methods. In response, technology-based interventions have emerged as promising tools, offering reliable and versatile means to monitor and understand various aspects of ASD, including physiological functions, behaviors, and emotions [8].

Consequently, in the field of ASD and wearable technology, several systematic reviews have made significant contributions. Cabibihan et al. [9] were among the first to categorize technologies like eye trackers, movement trackers, and speech detectors, assessing their effectiveness and limitations. They found that many were in early development stages, facing challenges in clinical testing. Similarly, Wali and Sanfilippo [10] reviewed assistive technologies in the workplace, highlighting strengths and limitations while emphasizing a research gap that requires further refinement. In the same way, Ahuja et al. [11] focused on wearables for monitoring behavioral and physiological responses in children, noting their potential for personalized interventions. Also, Black et al. [12] reviewed wearables for functional skills in youth with ASD, stressing the need for more research due to limited evidence. In a similar study, Welch et al. [13] explored the integration of wearables with AI in psychiatric care, particularly for ASD, but noted the lack of randomized controlled trials. Similarly, Francese and Yang [14] examined wearable devices and machine learning for early ASD detection, pointing out limitations like small sample sizes and insufficient real-world validation. Furthermore, Anis and Mohd [15] summarized the role of assistive technology in supporting communication, social interaction, and cognitive skills in individuals with ASD, but highlighted the need for more comprehensive studies. Koumpouros and Kafazis [16] discussed challenges like small sample sizes, privacy concerns, and the need for practical, customizable solutions in wearable and mobile technologies for ASD interventions. Likewise, Gao et al. [17] categorized wearable technology into signal acquisition and interactive feedback systems, noting their potential but also the need for clinical validation. Taj-Eldin et al. [18] reviewed wearable technologies for monitoring ASD, covering various sensors and devices for physiological activity, facial expression, behavior, and sensory processing. Their findings underscored the strengths and limitations of these technologies, while also identifying areas for further research to improve their accuracy and applicability in clinical and everyday settings, though they did not address newer devices that have emerged recently.

In contrast, our review provides a comprehensive and updated analysis of wearable technologies for ASD, aimed at a multidisciplinary audience, including clinicians, researchers, and technology developers in the field of ASD management. The primary goal is to provide insights into how wearable technologies can assist in the ongoing management and support of individuals with ASD. While many of the wearables discussed may appear more suited to clinical diagnostics or specific interventions, they offer substantial potential for daily use in personalized ASD care. It is important to note that many of the wearables analyzed in this review were not necessarily tested specifically in the ASD context, but they are related to variables that are highly relevant to ASD management, such as emotional regulation and sensory processing.

Unlike previous reviews, such as that by Taj-Eldin, which employed a primarily narrative methodology, our approach is systematic and rigorous. We utilize clearly defined inclusion and exclusion criteria, a PRISMA-based selection process, and quantitative data analysis to ensure methodological robustness. Our review addresses gaps identified in previous studies by incorporating emerging and advanced technologies that were not covered in earlier reviews. Specifically, we include eye-tracking devices, posture, and balance sensors, augmented reality technologies, and sleep-monitoring devices. Additionally, we enhance the categorization of wearables by focusing on specific ASD domains, including executive function, psychomotor skills, and the behavioral/emotional/sensory (BES) domain. This refined approach offers a more detailed and context-specific evaluation of how wearable technologies can address various ASD-related needs, thereby providing a more comprehensive and updated perspective of the field. Therefore, this scoping review aims to systematically assess the current landscape of commercial wearable technologies for ASD, identify gaps in clinical validation, and suggest future research directions. The analysis is organized across three key domains and categorized by the types of sensors used in these technologies.

The paper is structured as follows: Section 2 discusses the physiological variables relevant to ASD that are manageable by wearable technologies, providing an overview of how different sensors are used to track these variables. Section 3 outlines the methodology used in conducting this scoping review, including the inclusion criteria and data collection process. Section 4 presents the findings, organized by three domains and sensor types, offering insights into the current state of wearable technologies. Section 5 discusses the implications of the findings, addresses limitations, and proposes directions for future research. Finally, Section 6 provides the conclusions, summarizing the key takeaways from the study and their broader significance in the field of ASD management.

## 2. Physiological Variables in ASD

**Figure 1:**
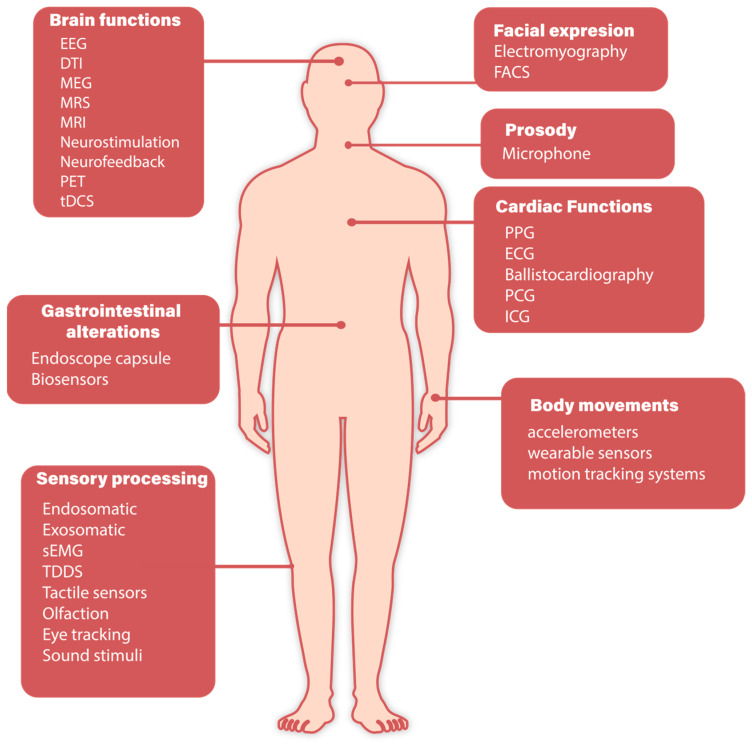
Diagram of the main body functions that can be supported by advanced diagnostic technologies in individuals with ASD.

The management of physiological variables in individuals with ASD has benefited considerably from advances in wearable device technologies. These devices facilitate the real-time acquisition of a diverse array of biological signals, which are instrumental in comprehending and addressing the multifaceted dimensions of ASD. The Figure 1 highlights the key body functions that can be supported by these technologies, including brain functions, body movements, cardiac functions, facial expressions, sensory processing, gastrointestinal alterations, and prosody. Advanced techniques such as electroencephalography (EEG), functional near-infrared spectroscopy (fNIRS), electromyography (EMG), and biosensors, among others, are employed to capture these functions, providing a comprehensive assessment of physiological and behavioral states.

This figure serves as a visual guide to understanding the complex physiological interactions that these devices aim to manage, providing the necessary context for the subsections that follow, where the specific applications of each technology in supporting the management of ASD are explored in detail.

### 2.1. Body Movement

Body movement, spanning from fine to gross motor skills [19], is analyzed through techniques such as wearable sensors and motion-tracking systems [9]. Wearable sensors, which include accelerometers, measure motion acceleration in different axes, thus obtaining velocity and displacement over time. While they offer convenience and portability, wearable sensors can compromise data quality in aggressive scenarios. Motion tracking systems utilize cameras, sometimes in conjunction with sensors, to capture and analyze movement patterns, thereby aiding in the quantification and characterization of motor behavior. These methods contribute to the study of motor deficits in ASD, encompassing issues with postural stability, manual motor functions, and anticipation [20]. Motor difficulties in ASD, which remain consistent from early childhood, are closely associated with the core symptoms of ASD.

### 2.2. Brain Function

A variety of methods are used to investigate the brain, including those aimed at understanding the structural features of the brain, using techniques such as MRI and diffusion tensor imaging (DTI). Another approach involves neurotransmitter assessment using techniques such as magnetic resonance spectroscopy (MRS), positron emission tomography (PET), and single-photon emission computed tomography (SPECT). In addition, neurostimulation methods such as transcranial direct current stimulation (tDCS), vagus nerve stimulation (VNS), and transcranial magnetic stimulation (TMS) are employed. Investigation of the magnetic fields generated by neuronal activity is also carried out using techniques such as magnetoencephalography (MEG) and magnetic resonance spectroscopy (MRS). Finally, the analysis of brain electrical activity employing EEG is used. Likewise, the techniques used to study these variables can be classified into two categories: imaging techniques (MRI, DTI, MRS, PET, SPECT) and electrical signal techniques (tDCS, TMS, MEG, EEG). Imaging techniques provide insight into the structural characteristics of the brain and its neurotransmitters. MRI and DTI reveal alterations in brain morphology and connectivity patterns in individuals with ASD) [21,22], while MRE, PET, and SPECT reveal alterations in neurotransmitter systems such as glutamate, GABA, dopamine, serotonin, and acetylcholine associated with cognitive and behavioral deficits observed in ASD [23]. Electrical signaling techniques modulate neuronal activity within the central nervous system and investigate magnetic fields and electrical activity in the brain. tDCS and TMS show promise as therapeutic interventions for ASD by restoring excitatory–inhibitory cortical balance [24] while MEG and EEG detect abnormalities in brain activity patterns and alterations in neuronal connectivity in individuals with ASD, guiding therapeutic interventions such as neurofeedback (NFB) therapy aimed at addressing associated abnormalities [25,26]

### 2.3. Cardiac Functions

Cardiac function encompasses a multitude of physiological variables indicative of cardiovascular health and autonomic nervous system regulation. Among these variables are blood volume pulse (BVP), blood pressure (BP), heart rate (HR), heart rate variability (HRV), and respiration rate (RR). BVP, as a dynamic signal reflecting changes in blood volume beneath a sensor, is assessed primarily through photoplethysmography (PPG) techniques [27]. Alterations in BVP have been implicated in ASD, particularly in individuals experiencing sleep disturbances, suggesting its potential utility as a surrogate measure for identifying comorbid conditions [28]. BP, representing the force exerted by circulating blood against arterial walls, comprises systolic blood pressure (SBP) and diastolic blood pressure (DBP) values, with photoplethysmography utilized for non-invasive measurements [29]. Research indicates potential associations between altered BP regulation and autonomic dysfunction in ASD, highlighting physiological distinctions from neurotypical individuals [30]. HR, measured in beats per minute, provides insight into cardiac activity and is assessed through various techniques, including electrocardiography (ECG), ballistocardiography (BCG), phonocardiography (PCG), and impedance cardiography (ICG) [31]. In the context of ASD, HR monitoring alongside HRV assessment is employed to investigate autonomic nervous system functioning and emotional regulation, with altered HR patterns associated with behavioral challenges [32]. HRV, indicative of the variability in time intervals between heartbeats, offers insights into sympathetic and parasympathetic nervous system activity, measured through techniques such as ECG, BCG, PCG, and ICG [33]. Individuals with ASD often exhibit lower HRV relative to neurotypical counterparts, suggesting potential autonomic dysfunction and providing a potential biomarker for understanding the underlying physiological mechanisms of the condition [34]. Lastly, RR, denoting the number of breaths per minute, is a crucial parameter indicative of respiratory function, assessed via techniques such as ECG and PPG modulation schemes [35]. Altered autonomic function in ASD may manifest as dysregulation in RR alongside changes in other physiological parameters, emphasizing the relevance of RR monitoring in assessing respiratory health and autonomic function in this population [36]. These variables collectively provide a comprehensive framework for understanding cardiac function and its implications in the context of ASD.

### 2.4. Facial Expression

Facial expression is a crucial aspect of nonverbal communication, encompassing movements such as expressions, eye contact, and head orientation which are crucial for assessing human emotions and behaviors. Techniques such as EMG, facial action coding system (FACS) [37], human–robot interaction (HRI) systems [38] and fMRI [39] are used to analyze facial behavior. Thus, EMG measures the electrical activity of facial muscles and provides information about the muscle movements of expressions. FACS classifies facial movements into discrete action units, allowing detailed expression analysis. HRI systems use facial behavior analysis to improve the understanding of robots and thus have social interaction with people. In addition, fMRI plays a key role in elucidating the neural substrates underlying facial expression processing, shedding light on the neurobiological mechanisms of social cognition and emotional processing Notably, the recognition and production of atypical facial expressions are common in autism, which hinders social interaction, and understanding these deviations helps researchers to design interventions for social communication deficits.

### 2.5. Gastrointestinal Alterations

Commonly seen in ASD, gastrointestinal alterations involve digestive tract conditions leading to symptoms like abdominal pain, chronic diarrhea, and constipation [40]. Research links gut microbiota dysbiosis in ASD to neurological effects via neural, immune, and endocrine pathways. A dysregulated gut microbiota may affect neurotransmitter production and exacerbate ASD-related behaviors [41]. Techniques such as video capsule endoscopy (CE) [42] and bacterial biosensors [43] offer non-invasive methods to comprehensively assess GI alterations, providing detailed imaging and detecting biomarkers associated with inflammation and dysbiosis.

### 2.6. Prosody

Prosody is a linguistic concept that encompasses acoustic features such as pitch, rhythm, accent, and intonation, which convey meaning and emotion in speech. Traditional methods, such as manual annotation by speech therapists, and modern approaches using machine learning models trained on speech data are used to analyze prosody [44]. Traditional methods rely on auditory observation and subjective judgment, whereas machine learning models provide objective assessments by automatically extracting and analyzing acoustic features from recorded conversations. Studies on ASD reveal anomalies in prosody, such as monotone speech and inappropriate accents [45]. Machine learning models, particularly those focusing on the pitch, excel at discerning prosodic deviations compared to speech therapists. Interventions targeting prosody show promise in promoting typical prosodic patterns in individuals with ASD.

### 2.7. Sensory Processing

Sensory processing refers to the neurological mechanisms involved in receiving, interpreting, and responding to sensory stimuli from the environment. It encompasses a spectrum of sensory modalities through which individuals perceive and interact with their surroundings. The understanding of sensory processing abnormalities holds particular significance in the context of ASD, where individuals often exhibit atypical responses to sensory stimuli. Electrodermal activity (EDA) serves as a physiological marker of sympathetic nervous system (SNS) arousal [46]. EDA assessment involves techniques such as endosomatic and exosomatic measurements utilizing specialized electrodes, offering insights into sympathetic activity variations. Studies have extensively investigated EDA measures in individuals with ASD to elucidate sensory processing abnormalities [47]. Muscle response, essential for movement and stability, is studied through surface electromyography (sEMG), providing insights into muscle function during movements [48]. Individuals with ASD may experience motor skill difficulties, and interventions incorporating play have shown promise in improving fine motor skill development [49]. Transdermal drug delivery systems (TDDSs), offering a non-invasive approach to medication administration, present a potential avenue for managing ASD-related symptoms [50]. Touch sensing interventions, utilizing wearable systems with tactile sensors, aim to improve tolerance to physical contact and promote social interaction among individuals with ASD [51]. Olfaction, the sense of smell, is assessed through physiological measurements and neuroimaging methodologies, revealing alterations in individuals with ASD and highlighting the potential of olfactory dysfunction as a biomarker for diagnosis and characterization [52]. Eye movement analysis, facilitated by techniques like corneal reflection eye tracking, elucidates aberrant gaze behaviors characteristic of ASD, aiding in early diagnosis and intervention strategies [53]. Auditory processing studies, employing experimental paradigms and innovative technologies, explore atypical responses to sound stimuli among individuals with ASD, offering insights into sensory processing differences within the auditory domain [54]. In summary, these sensory modalities and associated techniques provide a comprehensive framework for understanding and addressing sensory processing challenges in individuals with ASD, thereby informing targeted interventions and personalized rehabilitation strategies.

Understanding the multifaceted nature of ASD requires a comprehensive examination across various domains of functioning. Executive function, encompassing cognitive processes such as planning, decision-making, and impulse control, plays a crucial role in the daily lives of individuals with ASD. This domain involves investigating structural characteristics of the brain, electrical activity, neurotransmitter levels, and the effects of neurostimulation on executive functioning. In parallel, the psychomotor domain focuses on motor skills, body movement patterns, and physiological responses, including heart rate variability and blood pressure, which are often affected in individuals with ASD. Additionally, the BES domain delves into the complex interplay of gastrointestinal alterations, emotional regulation, sensory processing differences, and their impact on behavior. The decision to integrate behavioral, emotional, and sensory elements into a single domain is based on the interconnectedness of these elements in ASD. Wearable technologies, which frequently monitor physiological parameters such as heart rate, electrodermal activity, and movement patterns, provide information that interconnects these three elements. For instance, emotional dysregulation is frequently triggered by sensory sensitivities and can manifest in behavioral problems. This makes it practical to study these elements together using integrated wearable systems. This unified approach reflects the actual experiences of people with ASD, where sensory stimuli often evoke emotional and behavioral responses that are difficult to separate. The accompanying Table 1 outlines the variables and techniques utilized across these domains to gain insights into the diverse facets of ASD. This multidimensional approach facilitates a more holistic understanding of ASD, aiding in the development of tailored interventions and support strategies.

In this review, we use the term ‘techniques’ in a broad sense to encompass both the methodologies and the sensors that are integral to the management and analysis of various physiological and behavioral parameters in individuals with ASD. While traditionally, ‘techniques’ refer to specific methods employed in data collection and analysis (such as fMRI or EEG), we recognize that, in the context of wearable technologies, the line between ‘technique’ and ‘sensor’ often becomes blurred. For example, methods like EEG not only describe a technique for measuring brain activity but also imply the use of specific sensors embedded in wearable devices to collect that data. The convergence of hardware (sensors) and methodologies (techniques) is particularly evident in the field of wearable technology, where the functionality of a device is dependent on the seamless integration of both. Thus, to streamline our discussion and avoid unnecessary repetition, we have chosen to use the term ‘techniques’ inclusively. This approach acknowledges that, in many cases, the sensor (e.g., accelerometer, ECG) is an inseparable part of the technique used to gather and interpret data. By adopting this inclusive terminology, we aim to provide a coherent narrative that reflects the interconnected nature of modern wearable technologies and their application in ASD research and management. This decision also reflects the practical realities of the field, where the focus is often on the combined efficacy of the sensor and the technique, rather than on maintaining a strict distinction between the two. We hope that this approach will provide clarity and make the review more accessible to readers who are interested in the application and outcomes of wearable technologies, without requiring extensive technical knowledge of the underlying components.

## 3. Methods

This review focuses on identifying existing wearable technologies and exploring those that could be utilized for the management of various ASD-related variables. These variables include structural characteristics of the brain, electrical activity in the brain, neurotransmitters in the brain, neurostimulation, prosody, body movement, muscle response, heart rate variability (HRV), blood volume pulse (BVP), blood pressure (BP), respiratory rate (RR), gastrointestinal alterations, heart rate (HR), electrodermal activity (EDA), touch sensing, olfaction, auditory response, and facial expression. The review follows the guidelines of the PRISMA 2020 [55] statement to ensure proper organization and clarity of its results.

**Inclusion and Exclusion Criteria**. Initially, we obtained 17,380 search results from various databases. The inclusion criteria for this review encompassed studies that (1) included participants with ASD, (2) employed wearable devices to manage ASD-related variables, (3) were published in English, and (4) had a publication date within the last 10 years (2014–2024). This ensured the relevance of the information. On the other hand, we excluded studies that (1) did not focus on ASD exclusively, (2) were from non-peer-reviewed sources, (3) were letters or reports, (4) were systematic reviews, meta-analyses, conference proceedings, or symposia, (5) were not primary studies, or (6) the full text was not available.

**Information Sources**. To determine the databases for the review, we grouped the keywords in our research questions into two categories: healthcare and computing technology. The healthcare databases included Biomed Central, PubMed, PubMed Central, JMIR, Nature, Springer Link, Wiley, and Taylor and Francis. The computing technology databases comprised IEEE Xplore, IOP Science, Inderscience, ScienceDirect, and MDPI.

**Search Strategy**. The search strategy involved combining keywords with Boolean connectors to limit the search results. The keywords were derived from the key concepts that are part of the research questions. The primary search query used was as follows:

*(“autism” OR “ASD”) AND “wearable” AND (“brain” OR “Neurostimulation” OR “Prosody” OR “Body movement” OR “Muscle response” OR “HRV” OR “HR” OR “BVP” OR “BP” OR “RR” OR “Gastrointestinal” OR “Electrodermal” OR “Touch” OR “Olfaction” OR “Auditory” OR “Prosody” OR “Facial expression”)*

For ScienceDirect and MDPI, the keywords were split into smaller groups due to search limitations. The searches on ScienceDirect were conducted in separate segments:

*(“autism” OR “ASD”) AND “wearable” AND (“brain” OR “Neurostimulation” OR “Prosody” OR “Body movement” OR “Muscle response”), (“autism” OR “ASD”) AND “wearable” AND (“HRV” OR “HR” OR “BVP” OR “BP” OR “RR”), (“autism” OR “ASD”) AND “wearable” AND (“Gastrointestinal” OR “Electrodermal” OR “Touch” OR “Olfaction” OR “Auditory”), and (“autism” OR “ASD”) AND “wearable” AND (“Prosody” OR “Facial expression”).*

**Figure 2:**
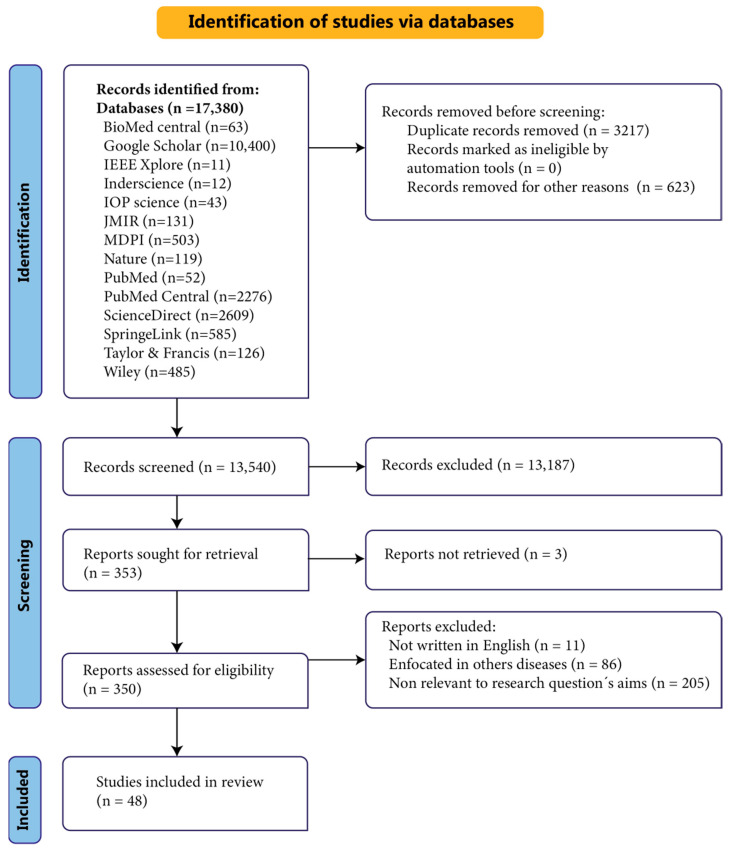
PRISMA flow diagram of the search strategy.

For MDPI, the search was conducted with specific terms focusing on each variable. **Selection Process.** Our study initially identified 17,380 relevant papers based on their titles and abstracts. Subsequently, we retrieved data from various databases, yielding the following results: Biomed Central (63), Google Scholar (10,400), IEEE Xplore (11), IOP Science (43), Inderscience (12), JMIR (131), MDPI (503), Nature (119), PubMed (52), PubMed Central (2276), ScienceDirect (2609), SpringerLink (585), Taylor and Francis (126), and Wiley (485). Following this, five subject matter experts (SMEs) conducted a thorough screening of each work and categorized the data accordingly. During the initial review process, papers were excluded based on predefined inclusion and exclusion criteria. The remaining studies underwent a comprehensive analysis focusing on their research objectives and questions, resulting in the identification of 48 studies that met all inclusion criteria. A PRISMA-based diagram illustrating our search strategy is presented in Figure 2.

**Data Collection and Analysis**. During this phase, a structured approach was employed to organize information obtained from the review of the 48 primary studies. Oversight of the analysis was provided by three SMEs, who systematically extracted pertinent details regarding wearable devices utilized to manage variables in individuals with ASD. Information of interest encompassed the targeted variable, brand, model, and type of wearable device, as well as key device features, operating mechanism, sensor(s) employed, and the Food and Drug Administration (FDA) status. This methodology facilitated a comprehensive evaluation of wearable technologies pertinent to ASD detection and treatment.

## 4. Results

### 4.1. Study Selection

The selection of studies for this review was conducted through a rigorous and systematic approach, following the PRISMA framework. An initial comprehensive literature search across databases such as Biomed Central, PubMed, IEEE Xplore, and ScienceDirect resulted in 17,380 records. As depicted in Figure 2, these records were distributed according to their database of origin. The first phase of screening involved the elimination of 3217 duplicate records and 623 records unrelated to ASD, resulting in 13,540 records. These were then further screened based on titles and abstracts, leading to the exclusion of 13,187 records. Subsequently, 353 records were subjected to a full-text evaluation to assess their eligibility. Studies were required to meet specific criteria, including (1) involvement of participants with ASD, (2) use of wearable devices for the management of ASD-related variables, (3) publication in English, and (4) publication within the last ten years (2014–2024). Exclusion criteria included studies that did not exclusively focus on ASD, were non-peer-reviewed or fell into categories such as letters, reports, systematic reviews, meta-analyses, conference proceedings, and symposia. Additionally, studies without available full texts were excluded. This meticulous selection process culminated in the exclusion of 305 studies, resulting in a final set of 48 studies deemed appropriate for inclusion in the analysis.

### 4.2. Study Characteristics

The analysis of the 48 selected studies began with an a priori coding process. We categorized each study based on the specific ASD variables they supported and the wearable technologies they involved. This initial step helped us organize the studies into key domains related to ASD management, such as executive function, psychomotor skills, behavioral and BES, and sensory processing. During this process, we identified the sensors and physiological parameters that were monitored, such as HRV, brain activity, muscle movement, and emotional responses. In cases where studies explicitly mentioned wearable devices, we recorded the specific technology and its use. However, for many studies that did not explicitly mention wearable devices, we inferred the potential use of such technology based on the study’s methods and the physiological parameters being tracked. This inference was based on our knowledge of existing wearable technologies and their relevance to the areas being studied. After completing the a priori coding, we conducted a web search to verify and identify the wearable devices that could be linked to the support needs described in the studies. This search helped us match the inferred wearable technologies with available or emerging devices, ensuring our analysis reflected the current state of wearable technology in ASD research.

## 5. Commercial Wearables for ASD

### 5.1. Executive Function Domain

The executive function domain encompasses a wide array of functions, including memory, attention, language processing, and problem-solving skills, all of which are integral to understanding and addressing ASD. In the pursuit of more effective interventions and therapies, researchers and clinicians have turned to innovative technologies, such as wearable devices, to gain insights into the cognitive processes underlying ASD. Table 2 provides an overview of commercially available wearable devices tailored specifically for studying cognitive aspects of autism, highlighting their features, sensor technologies utilized, and regulatory support statuses. These devices offer promising avenues for exploring the intricate nuances of cognitive functioning in individuals with ASD, potentially paving the way for personalized interventions and improved outcomes.

### 5.2. Psychomotor Domain

The psychomotor domain involves the coordination between mental processes and physical actions, which is particularly significant for individuals with ASD who may experience difficulties with motor skills and movement control. Advances in wearable technology offer innovative solutions for monitoring and enhancing psychomotor skills by providing real-time, detailed insights into motor performance and muscle activity. These devices, equipped with various sensors, enable comprehensive tracking and analysis of psychomotor functions. An examination of the different wearable devices suitable for psychomotor assessment is detailed in Table 3, which outlines the main features and sensor technologies of each device. This overview highlights the diverse capabilities of these wearables, reflecting their potential to contribute significantly to the evaluation and management of psychomotor skills.

### 5.3. Behavioral/Emotional/Sensory Domain

The behavioral/emotional/sensory domain encompasses a range of activities and responses that can be influenced by various external and internal stimuli, which is particularly relevant in the context of ASD. This condition is characterized by a spectrum of behavioral challenges, including difficulties in sensory processing, social interaction, and adaptive functioning. Wearable technologies have emerged as a promising tool for monitoring and managing these BES aspects, offering novel ways to collect real-time data and provide targeted interventions. These devices, equipped with various sensors, can capture physiological and behavioral data that are crucial for understanding and addressing the needs of individuals with ASD. Table 4 presents a detailed overview of wearable devices that have been explored for their potential applications in BES monitoring. These devices range from minimally invasive capsules for internal visualization to non-invasive wearable sensors designed for sensory modulation. By integrating data from such devices, researchers and clinicians can gain deeper insights into the behavioral patterns associated with ASD and tailor interventions more effectively.

## 6. Discussion

In this section, we explore the application of wearable technology in the management of ASD. We begin by analyzing how wearable devices provide information on symptom patterns across different levels of severity. Next, we discuss the diversity of wearable device form factors and address regulatory considerations by examining the FDA approval status of these devices. Furthermore, we delve into the types of sensors utilized in wearable devices across various domains, including executive function, psychomotor control, and BES. This is followed by a discussion of emerging solutions and, finally, an examination of the limitations of this work.

To effectively analyze symptom patterns, it is essential to consider the current diagnostic framework for ASD. In the DSM-5 (Diagnostic and Statistical Manual of Mental Disorders), previous autism subtypes such as Asperger’s Syndrome and Pervasive Developmental Disorder Not Otherwise Specified were consolidated into a single diagnosis of ASD, categorized into three severity levels. Sung et al. [170] noted that this shift from DSM-IV-TR to DSM-5 prioritized symptom severity and support needs, ranging from minimal (level 1) to very substantial (level 3) support. Gardner et al. [171] emphasized that this new approach reflects the wide variability in how ASD symptoms present in daily life, aiming to provide more personalized diagnostic guidelines to guide therapeutic interventions. The DSM-5 diagnostic criteria for ASD focuses on two main domains: social communication difficulties and repetitive or restrictive behaviors. While traditional assessments rely on interviews and standardized tests, wearable technology adds precision by capturing continuous physiological data in daily life settings. According to Alhassan et al. [172], devices that track brain activity EEG, HR, and EDA provide insights into symptom patterns that may not be observable during clinical evaluations, enhancing the understanding of symptom variability.

For individuals with severity level 1, who exhibit milder symptoms, wearables can detect subtle physiological changes, such as stress responses, which might not be noticeable in clinical settings. Taj-Eldin et al. [18] suggested that devices monitoring HR and HRV could offer valuable data on cognitive self-regulation challenges, helping clinicians design more personalized interventions.

In cases of severity level 2, where social communication difficulties and self-regulation challenges are more pronounced, wearable devices that monitor EDA and HR can provide key insights into the individual’s emotional state. For example, the Empatica EmbracePlus, which tracks EDA, can detect increased emotional arousal that might trigger repetitive behaviors or emotional crises. This is especially valuable in understanding variability in self-regulation across different environments.

For individuals with severity level 3, who often require continuous support due to severe communication and behavioral challenges, physiological monitoring is essential for identifying factors that exacerbate or alleviate symptoms. Gastrointestinal issues are common among individuals with severe ASD, and research has shown a significant association between an altered gut microbiota and these symptoms. Xu et al. [173] highlighted that individuals with ASD often exhibit an imbalanced composition of gut bacteria, which may contribute to both gastrointestinal and behavioral issues. While gastrointestinal symptoms are not part of the DSM-5 diagnostic criteria, devices like the GutTracker can monitor digestive functions, providing clinicians with additional insights into factors contributing to distress and behavioral dysregulation. This kind of monitoring, paired with the growing understanding of gut–brain interactions, may help identify links between physiological discomfort and emotional crises, offering a more holistic approach to treatment.

Eye-tracking technologies, such as Tobii Pro Glasses 3, also play a significant role in understanding how individuals with ASD process social cues. Alhassan et al. [172] mentioned that these devices can reveal difficulties in interpreting non-verbal communication, particularly in individuals with level 3 severity, who struggle more with social interactions. Additionally, sensory modulation devices, like the Apollo Neuro, which uses gentle vibrations or calming sounds, are beneficial for managing responses to overwhelming sensory stimuli. These tools can be particularly useful for preventing sensory overload, a key trigger for emotional crises in individuals with sensory hyperreactivity.

**Figure 3:**
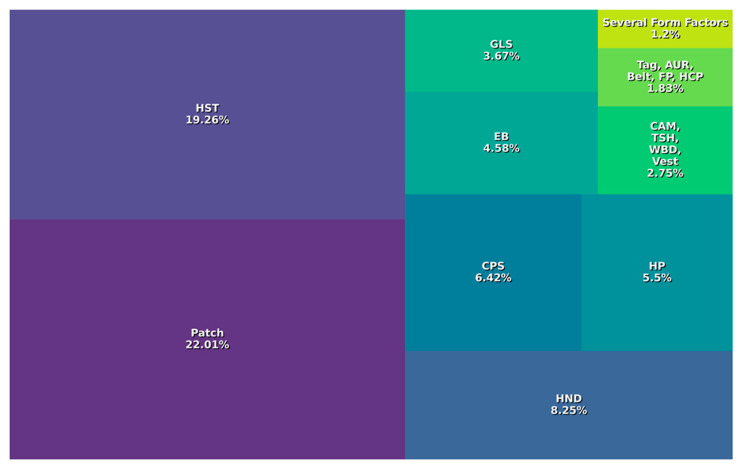
Frequency of wearable form factor types used. HST: headset; HND: handheld; CPS: capsule; HP: headphone; EB: earbuds; GLS: glasses; CAM: camera; TAG: tag; TSH: t-shirt; WBD: wristband; VST: vest; AUR: auricular; BLT: belt; FP: force plate; HCP: headcap; several form factors relates to armband, smartband, chest belt, headband, grid, shoe pod, shorts, and sleeve, each with 1.2%.

Regarding form factor, Figure 3 shows a breakdown of various wearables by form factor, excluding smartbands, smartwatches, and rings due to the large number of models in these categories. This exclusion allows for a more detailed analysis of less common wearables, providing valuable market data. Although excluded due to their variety of functionality and styles, future research could examine these categories separately to identify trends and preferences in specific subtypes. However, this exclusion should not underestimate their usefulness in the ASD field, as these devices offer advanced monitoring and tracking features, such as task reminders, fitness tracking, and instant communication, which can be beneficial to individuals with ASD and their caregivers. The data in this figure show that patches and headsets are the most common formats among wearable devices, accounting for 20.4% and 19.4% of the total, respectively. This dominance may be due to their versatility, convenience, and wide range of applications in a variety of settings. Patches have a wide range of applications and are mainly used to monitor movement and vital signs, and can also be used to track intestinal movements. Headsets, on the other hand, provide hands-free operation for activities that primarily involve reading EEG signals. Handheld, capsule, and headphone devices occupy secondary but notable positions in the portable device landscape. Handheld devices are primarily used to analyze ECG-type signals. Capsules, on the other hand, are used for intestinal observation and monitoring using camera-type sensors. As for headphones, in addition to their primary function of audio playback, they also incorporate additional capabilities such as heart rate and body temperature measurement. Wearables such as glasses, cameras, and clothing such as T-shirts and vests, although less common, show a moderate representation in the wearable device landscape, suggesting their potential utility for users with specific needs. Glasses, potentially equipped with augmented reality displays or cameras, have diverse applications, including eye tracking and the development of augmented and virtual reality technologies, which are particularly useful in the case of ASD. Less commonly, cameras are used for body movement analysis and eye tracking. Wearable devices, such as T-shirts and vests, are equipped with sensors for biometric monitoring and activity tracking and can be easily integrated into daily activities. The next segment of wearable devices includes several form factors, each targeting specific areas. These include armbands, headsets, belts, force plates, helmets, and tags designed for specific use cases or industries. Wristbands are similar to smartwatches but with a focus on comfort. Belts focus on motion analysis using sensors such as IMU, while force plates are dedicated to gait and posture analysis. Headcaps, similar to headsets, are used in neuroscience research or the development of brain–computer interfaces, but with a different design. Less common form factors include armbands, chest straps, ear clips, grids, headbands, shoe pods, shorts, and sleeves. The reasons for their low adoption may be diverse, such as comfort limitations, lack of accuracy in data collection, or limitations in the applicability of these devices in certain contexts.

Thus, Figure 3 shows the diversity of wearables, with a variety of form factors to meet different needs and preferences. However, it is imperative to consider the relationship between form factors and the specific needs of users, particularly those with ASD. The exploration of less common formats of wearable devices may have additional benefits in terms of adaptability and customization to the individual needs of people with ASD. This could increase the acceptability and effectiveness of these devices in use. Consequently, there is a significant opportunity to research and develop wearables with less conventional formats that may offer more effective and personalized solutions for this specific community.

**Figure 4:**
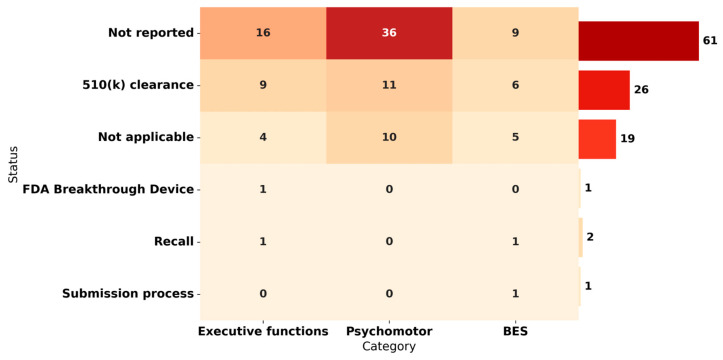
FDA status for wearable devices in ASD domains.

The approval of an institution, particularly the FDA, is crucial to ensuring the safety and efficacy of medical devices, especially in sensitive areas such as ASD. The FDA plays a critical role in evaluating and regulating medical devices to ensure their quality and reliability. In this study, we analyzed the approval status of several wearable devices that could be used in the context of ASD, although they were not specifically designed for this purpose. The various headings in Figure 4 provide information on the regulatory status of each device. “Not reported” indicates that the approval status has not been reported. “510(k) clearance” indicates that the device has received FDA clearance through the 510(k) process, which evaluates the safety and effectiveness of devices that are “substantially equivalent” to previously cleared devices. “Not applicable” indicates that FDA regulation does not apply to this particular device. “FDA Breakthrough Device” refers to devices that offer significant advantages over existing methods and have received special designation from the FDA to expedite their development and review. “Recall” indicates that the device has been withdrawn from the market due to safety or effectiveness concerns. “Submission process” indicates that the device is under review by the FDA. The results show that most devices do not have their approval status reported, which may raise questions about their suitability for use in the ASD setting. Devices with FDA Breakthrough Device approval are the least common, with only one device found, suggesting that there is a lack of devices that provide more effective treatment than the standard of care for the treatment or diagnosis of human diseases or conditions such as ASD. We also note that most of the devices with “not reported” regulatory reports have a higher incidence in the psychomotor domain, followed by executive functions, while BES has a relatively lower representation. On the other hand, devices with “510(k) clearance” have a more balanced distribution among the three domains. However, it is important to note that the total number of devices in this category is lower compared to the “not reported” reports. In addition, there is a limited presence of devices in the “Not applicable”, “FDA Breakthrough Device”, “Recall”, and “Submission process” categories. It is important to note that several wearables may have certifications other than FDA, such as CE marking for marketing in the European Union or ISO certification for international quality standards.

**Figure 5:**
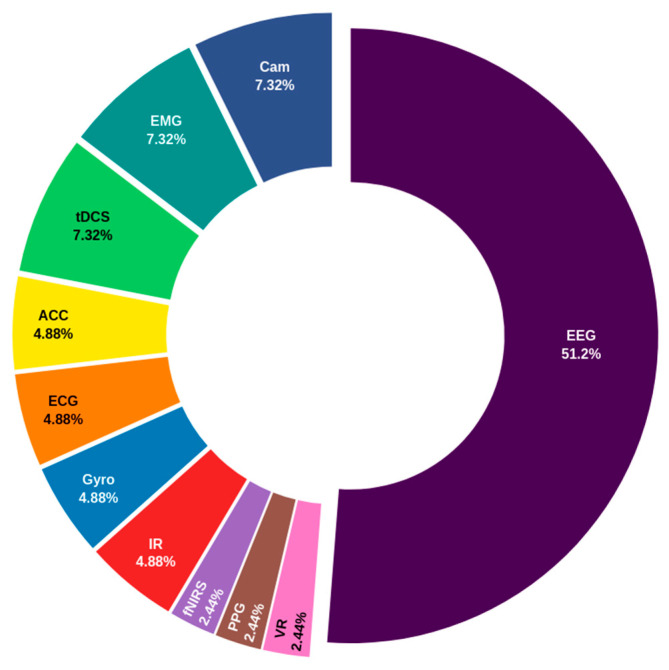
Distribution of commercially utilized sensors for executive function management in autism. The chart highlights the predominance of electroencephalogram (EEG) over other technologies such as transcranial direct current stimulation (tDCS), electromyography (EMG), and cameras (Cam). It also shows the use of less prevalent sensors like accelerometer (ACC), electrocardiogram (ECG), gyroscopes (Gyro), infrared (IR), functional near-infrared spectroscopy (fNIRS), photoplethysmography (PPG), and virtual reality (VR), indicating the diversity of tools employed in this domain.

Figure 5 depicts the outcomes of the sensors utilized in commercial applications within the domain of executive function for autism management. The graph illustrates the preponderance of the EEG, which plays a pivotal role in monitoring brain activity. This EEG dominance, which accounts for over 50% of usage, underscores the industry’s reliance on neurophysiological tools to assess higher cognitive functions that are often compromised in ASD. The preference for EEG reflects the paramount importance placed on obtaining precise neuroelectrical data in this context. Furthermore, the notable presence of tDCS and electromyography (EMG), both with 7.3%, indicates a trend towards the integration of technologies that not only record but also modulate brain activity and assess muscle response. This evolution suggests a shift towards a more holistic and personalized approach to interventions, beyond simple passive monitoring. The equal involvement of cameras concerning tDCS and EMG reflects the growing importance of behavioral capture in the assessment of executive function. This indicates an interest in methods that complement physiological data with direct observations, which may enhance the diagnostic accuracy and effectiveness of interventions. Conversely, the lesser prevalence of technologies such as accelerometers, ECG, and gyroscopes, with shares between 2.4% and 4.9%, indicates that, although valuable, these tools are more suited to specific or complementary applications compared to neurophysiological and behavioral sensors. Similarly, the lower participation of fNIRS, PPG, and VR, each at 2.4%, suggests that, while emerging and promising, these technologies have not yet achieved widespread commercial adoption for managing executive function in autism. This may indicate that their integration is still developing or is reserved for specialized cases.

**Figure 6:**
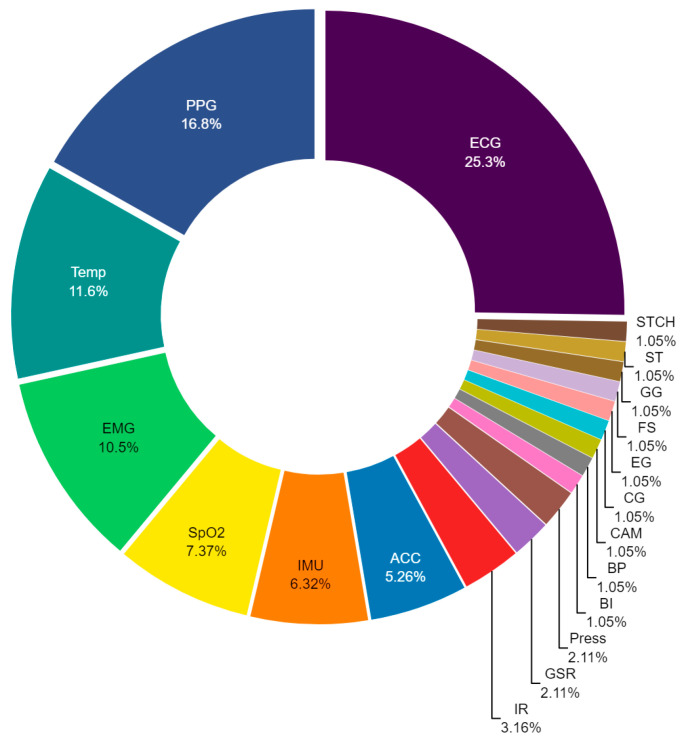
Distribution of commercially utilized sensors for psychomotor monitoring in autism. The chart highlights key sensors such as electrocardiogram (ECG), photoplethysmography (PPG), temperature (Temp), electromyography (EMG), blood oxygen saturation (SpO_2_), inertial measurement units (IMU), accelerometer (ACC), infrared sensor (IR), galvanic skin response (GSR), pressure sensors (Press), bioimpedance (BI), blood pressure (BP), cameras (CAM), capnography (CG), electrogoniometer (EG), footswitch (FS), galvanic gauge (GG), strain gauge (ST), and stretch sensor (STCH).

As illustrated in Figure 6, the distribution of sensors in the field of psychomotor monitoring in autism appears to prioritize the assessment of bodily functions that are directly observable and measurable, as opposed to the monitoring of executive functions, which tends to focus more on internal cognitive processes. The prominence of ECG and PPG indicates a significant focus on the cardiovascular system, suggesting that physiological responses such as heart rate variability and peripheral oxygenation are crucial for understanding arousal state and emotional regulation in individuals with autism. This approach has direct implications for the development of interventions that seek to stabilize the physiological state as a foundation for psychomotor improvement. Conversely, the incorporation of temperature, EMG, and SpO_2_ sensors, coupled with the diminished reliance on technologies such as IMU, ACC, and IR, indicates a shift towards the acquisition of physiological data rather than movement assessment perse. This could signify a tendency towards the monitoring of the physical consequences of psychomotor disorders rather than the comprehensive observation of movement itself. This indicates that interventions may be developed not only to rectify aberrant movements but also to address the underlying physiological responses that may be influencing those movements. The low percentage of use of sensors such as GSR (2.1%), Press (2.1%), and those represented in the group with a 1.05% prevalence suggests that these technologies are primarily employed in highly specific applications where additional precision or a particular focus on less common aspects of psychomotor monitoring is required. However, this same result also presents an opportunity for further research, as the low prevalence of these sensors could indicate underexplored areas with potential for future innovation. The identification of these gaps suggests that, although not common in current practice, these sensors could play a more significant role as new studies are developed that demonstrate their efficacy in the management of autism. This could lead to the development of therapeutic approaches that prioritize physiological regulation as a pathway to improving motor skills and quality of life for individuals with autism.

**Figure 7:**
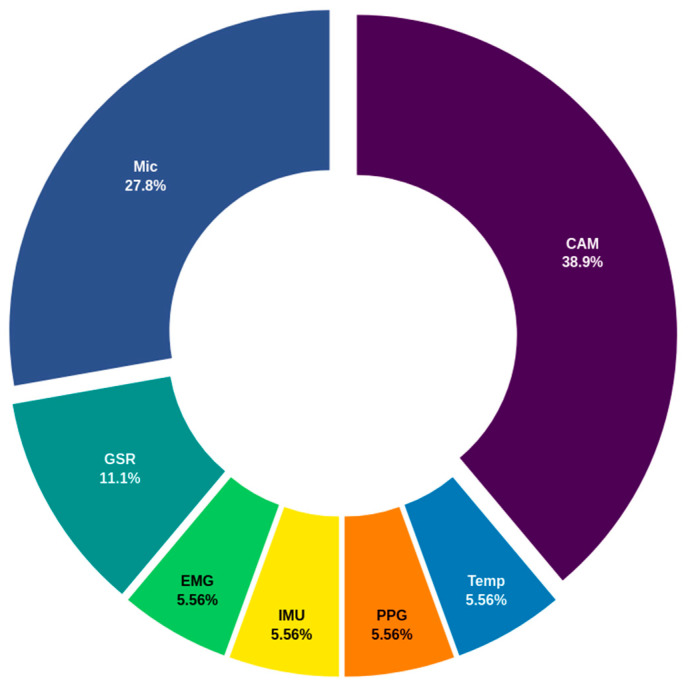
Distribution of commercially utilized sensors in the behavioral/emotional/sensory (BES) domain for autism. The chart highlights key sensors such as cameras (CAM), microphones (Mic), galvanic skin response (GSR), electromyography (EMG), inertial measurement units (IMU), photoplethysmography (PPG), and temperature (Temp).

Figure 7 provides an overview of the most commonly used sensors in the domain of BES monitoring in individuals with autism, indicating a lower diversity of technologies compared to other domains, such as psychomotor or executive. The prevalence of cameras and microphones, representing 38.9% and 27.8% of the total, indicates a strong focus on direct observation and auditory data capture. These tools are essential for the assessment of behaviors, emotions, and sensory responses, allowing for the analysis of facial expressions, postures, movements, and vocalizations, which is crucial for interpreting interactions and reactions in different contexts. The presence of the GSR sensor (11.1%) reflects the interest in measuring skin conductance as an indicator of emotional reactivity and stress, which are key aspects of emotional assessment within the autism spectrum. However, the lower prevalence of other sensors, such as EMG, IMU, PPG, and temperature sensors (each with 5.6%), suggests a more specialized focus on capturing physiological responses and movements that may be associated with emotional or sensory reactions. The limited diversity of sensors in this domain may indicate that current commercial solutions prioritize the direct and observable capture of BES responses, rather than integrating a broader range of physiological and motion data. This may indicate an area of potential for future technological development, whereby a more comprehensive and multidimensional assessment of behavioral, emotional, and sensory responses in individuals with autism could be achieved. Furthermore, the emphasis on a limited number of sensors may suggest that assessments in this domain are still in the early stages of technological advancement, thereby providing opportunities for future research that expands the use of other sensors and innovative approaches.

**Figure 8:**
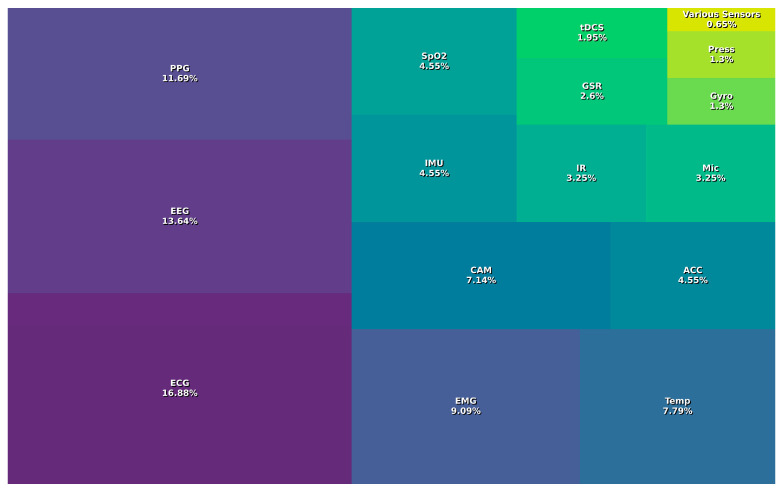
Frequency of sensors used in wearables. ECG: electrocardiogram; EEG: electroencephalogram; PPG: photoplethysmography; EMG: electromyography; CAM: camera; TEMP: temperature; SpO_2_: peripheral capillary oxygen saturation; ACC: accelerometer; IMU: inertial measurement unit; IR: infrared; MIC: microphone; GSR: galvanic skin response; tDCS: transcranial direct current stimulation; GYRO: gyroscope; PRESS: pressure; various sensors relates to bioimpedance, blood pressure, capnograph, electrogoniometer, fNIRS, footswitch, galvanometer, strain, stretch, and virtual reality, each with 0.6%.

Figure 8 presents a comprehensive overview of all domains, indicating that the most commonly used sensors, such as ECG (16.9%), EEG (13.6%), and PPG (11.7%), are critical for monitoring a range of physiological parameters. These sensors allow continuous measurement of heart rate, brain activity, and blood oxygenation, providing essential information for managing episodes of stress and anxiety, which are common in people with ASD. Changes in heart rate, for example, can indicate elevated levels of anxiety, allowing for early and personalized interventions. However, the implementation of these devices is not without its challenges. A major issue is the accuracy and reliability of the data collected. Sensors such as EEG and PPG must be properly calibrated and positioned to avoid erroneous readings that could lead to incorrect interpretations of emotional or physiological states. In addition, user comfort and acceptance are critical. Individuals with ASD may be sensitive to textures and pressures, which can make continued use of certain devices difficult. One solution to these challenges is the design of wearables that are more ergonomic and less invasive, using hypoallergenic materials and gripping techniques that minimize discomfort. Devices such as the Cumulus and the X.on EEG, with their lightweight and adjustable designs, are good examples of how design can positively impact the user experience.

Despite the wide range of sensors available for wearable devices, some sensors are underutilized. For example, pressure sensors and GSRs are used to a limited extent, although they could provide valuable data on the user’s stress and emotional response, which is particularly relevant in people with ASD who may have difficulty communicating their emotional states. Increasing the use of these sensors could help caregivers and therapists better understand and manage episodes of anxiety and stress in real-time. Similarly, the integration of underutilized sensors such as cameras and microphones (MIC) can complement physiological information with data on facial expressions and vocalizations, providing a more complete picture of emotional and behavioral state. Another underutilized sensor is the capnograph, which measures carbon dioxide levels in respiration and can be a critical indicator of respiratory well-being. Although more common in medical settings, its use in wearables could provide early warnings of respiratory problems, which is especially important for individuals with ASD who may also have associated respiratory conditions. The use of strain and stretch sensors is also limited. These sensors may be crucial for monitoring posture and movements and detecting repetitive or unusual behaviors that are characteristic of some individuals with ASD. In addition, these data could be used to develop intervention programs that promote better posture and reduce the risk of injury. Bioimpedance and blood pressure sensors are also rarely used in wearables for ASD. Bioimpedance can provide information on body composition and hydration status, while BP sensors can help monitor cardiovascular health. Incorporating these sensors could significantly improve comprehensive health monitoring in people with ASD. Finally, electrogoniometry and footswitch sensors could have interesting applications in gait and posture analysis. These sensors, although more common in biomechanical studies, could provide detailed data on movement patterns and help develop personalized physical interventions.

In terms of practical solutions, devices that integrate multiple sensors can offer a holistic approach to monitoring. For example, the Muse S (Gen 2), which integrates EEG, PPG, gyroscopes, accelerometers, and pulse oximetry sensors, enables comprehensive monitoring of brain activity, heart rate, and body movement. This is particularly useful for detecting early signs of stress or anxiety in people with ASD, as fluctuations in these parameters may precede a seizure episode. These combined data can help identify specific behavioral patterns, such as increased physical activity before an anxiety crisis, enabling proactive interventions.

The age-appropriateness of commercial wearables targeting executive function in individuals with ASD is often not explicitly defined, creating potential ambiguity regarding their suitability for younger users. B-Alert is one of the few devices that specifically mentions its suitability for individuals aged six and older, due to its ability to adjust in size. However, most devices lack clear age guidelines. For instance, the Meta Quest Pro, marketed as a high-end virtual reality headset with features such as eye and facial tracking, is primarily designed to enhance productivity and mixed-reality experiences. While its complex features and high price might suggest it targets adult users, the device does not explicitly prohibit use by younger individuals. Similarly, devices like Flow tDCS and LIFTiD, which focus on neurostimulation to improve cognitive performance, lack pediatric-specific evidence or guidelines. The absence of explicit recommendations for pediatric use suggests a lack of comprehensive validation for younger users, making it crucial to approach their use with caution in younger populations. While these technologies may offer benefits for improving executive function, their effects on developing brains, especially in children with ASD, are not well documented. This highlights a gap in research and product documentation, underscoring the need for more age-specific studies and guidelines to ensure the safety and efficacy of these tools across different age groups.

This uncertainty extends to devices used in the context of psychomotor assessment, where age guidelines are similarly unclear. Devices such as the Smart-Dx Evo and G-walk, commonly used for gait and movement analysis in clinical and sports settings, do not provide specific age recommendations, but are frequently employed across a wide range of patients, from those undergoing rehabilitation to athletes focused on performance enhancement. Likewise, devices like the Vital Patch RTM and Biobutton, primarily designed for continuous health monitoring in adults, particularly in cardiac care, are used in remote patient monitoring and hospital-to-home transitions. Although these devices do not explicitly exclude younger users, their suitability for children remains uncertain, and careful clinical oversight would be required when used in younger populations. Similarly, wearables like Astroskin and SmartShirt, which monitor vital signs in extreme or high-performance environments, are marketed towards adults, with pediatric-specific testing or guidelines again notably absent.

In the domain of BES management for ASD, many devices also lack clear pediatric guidance. For example, the PillCam SB 3, used primarily for gastrointestinal monitoring, explicitly states that it can be used by children as young as two years old, making it one of the few devices suitable for pediatric patients under medical supervision. In contrast, most emotional regulation and stress management devices, such as Apollo, Sensate 2, and Embr Wave 2, are aimed at adults seeking to improve their emotional well-being. These devices employ methods ranging from neurostimulation to temperature modulation, but they do not address the specific needs of younger users or provide safety data for children. This raises questions about their application in ASD populations, where emotional regulation is often critical. Similarly, assistive listening devices like IQbuds2 MAX, Neosensory Duo, and Olive Max are designed for hearing-impaired adults, yet may benefit individuals with ASD who experience sensory processing challenges. However, without pediatric testing or age-specific guidelines, their suitability for children remains uncertain. An example of a more versatile system is the Empatica Health Monitoring Platform, which is used to monitor physiological and emotional data. This platform offers a comprehensive system adaptable to both adults and youth with ASD, providing real-time data on emotional state and physical activity—crucial for behavioral management and the prevention of emotional crises. Despite their versatility, even platforms like this would benefit from more detailed pediatric-specific validation, reflecting the broader need for more age-appropriate research and guidelines across the wearable technology landscape.

By examining these wearables, it is evident that many lack clear age-specific guidelines, which is particularly problematic for managing ASD, as it can require intervention at any age—from early childhood to adulthood. Whether for diagnosis or treatment, the absence of detailed age recommendations limits the safe and effective use of these technologies across different age groups. Given the varying needs of individuals with ASD at different stages of life, there is an urgent need for more research and product specifications that ensure these wearables can be appropriately used for ASD management across all ages.

### 6.1. Challenges and Trends

Technology is changing the way we address the diverse needs of people with autism, particularly in areas such as executive function, psychomotor skills, and behavioral, emotional, and sensory regulation. However, there are challenges and trends that wearable technology for autism must address.

In perspective, several trends in wearable technology are emerging to tackle these areas. One significant trend is the enhancement of executive function, which often suffers in individuals with autism, impacting cognitive processes such as memory, planning, organization, and decision-making. Despite notable progress, wearables still need to integrate adaptive learning algorithms capable of adjusting assistance to the user’s changing needs and preferences over time. Furthermore, fully adaptive systems that continuously learn and adjust interventions accordingly are not yet commonplace. Integrating these wearable devices with other everyday life tools and platforms, such as calendars and educational applications, remains a challenge to create a unified system providing comprehensive support in all aspects of daily life.

Another important aspect is the improvement of psychomotor skills. Many autistic people have difficulties with fine and gross motor skills, coordination, and movement planning. The integration of advanced sensors into video game consoles can complement and enhance interventions. For example, motion sensors and haptic devices could be used to monitor and correct movements in real time, providing immediate and accurate feedback to the user. This not only improves the accuracy and effectiveness of motor therapies but can also make them more engaging and motivating for patients by incorporating elements of play and gamification. This is because video games have emerged as promising tools for improving cognitive function not only in healthy individuals [174] but also in those with specific disorders such as depression [175].

In the domain of BES regulation, wearables are advancing the delivery of real-time information and interventions to help people manage sensory sensitivities, emotional regulation, and behavioral issues. For example, augmented reality (AR) and virtual reality (VR) glasses are also emerging as valuable tools to address BES regulation. These tools equipped with facial recognition technology could provide real-time feedback on facial expressions during social interactions, helping people better understand and respond to social cues. Furthermore, biosensors integrated into wearable patches could detect physiological signals indicating stress or anxiety and prompt wearers to engage in calming activities or employ coping strategies.

Despite the diversity of sensors and functionalities in wearable devices, a significant portion lacks explicit mentions of specific applications for ASD, posing a challenge in tailoring wearables to meet the unique needs of individuals with ASD. Furthermore, there is a notable disparity in the availability and application of certain sensors compared to others, such as EEG and ECG sensors being common while others like GSR and bioimpedance sensors are less so. These underutilized sensors could provide valuable insights into the emotional arousal, stress levels, and physiological responses of people with ASD, essential for monitoring and understanding their condition. Another major challenge is the acceptability and comfort of the device for users with ASD. Many people on the autism spectrum have particular sensory sensitivities that may make certain devices uncomfortable or overwhelming. Devices such as chest patch biosensors and portable ECGs offer continuous monitoring capabilities; they should be designed to be unobtrusive, lightweight, and non-intrusive to avoid causing discomfort or anxiety. By prioritizing user comfort and sensory sensitivity considerations, wearable technology may be more accessible and beneficial for individuals with ASD. In the same way, devices such as smart shirts, vests, and belts offer continuous monitoring capabilities of biometric variables, such as heart rate, body temperature, and others. These devices could be especially useful for detecting and understanding physiological response patterns in people with ASD during different activities and situations without being as invasive or uncomfortable for people with ASD. One aspect is to improve interoperability between devices from different manufacturers, enabling more consistent data collection and better collaboration between various technologies. Another promising area is the use of these devices for active therapeutic interventions, not just passive monitoring. For example, the devices could be programmed to provide real-time feedback through vibrations, sounds, or lights when they detect signs of stress or specific behaviors, helping the user regulate their emotions and behaviors. Finally, while many current devices focus on adults or older children, it is critical to develop wearables suitable for infants and toddlers with ASD, allowing for early intervention and possibly improved long-term outcomes. The combination of these approaches would not only transform the way people with ASD are monitored and supported but would also open up new avenues for research and the development of innovative therapies. Therefore, collaboration with neurodiverse communities is essential to ensure that wearable technology meets the diverse needs of people with autism. By involving autistic people, caregivers, clinicians, and researchers in the design and development process, the industry can create truly effective, user-centered solutions.

### 6.2. Emerging Solutions

Emerging wearable solutions for ASD have revolutionized the diagnosis, treatment, and daily management of people with ASD, and future wearable devices tailored for ASD are expected to integrate cutting-edge technologies such as fMRI and advanced biosensors, as well as harness the power of the Internet of Things (IoT) and deep learning algorithms. First, wearable devices equipped with fMRI technology hold great promise for providing real-time information on brain activity patterns associated with ASD, as they could offer a non-invasive and continuous means of monitoring brain function in people with ASD, contributing to early diagnosis, personalized treatment planning, and tracking therapy efficacy. Furthermore, the integration of advanced biosensors into wearable devices tailored to ASD could enable comprehensive physiological and behavioral monitoring, as these biosensors, which use nanomaterials and nanotechnologies such as those currently used in the development of biosensors for healthcare, could detect subtle physiological signals and biomarkers associated with ASD symptoms, from monitoring stress levels to tracking sleep patterns and detecting changes in heart rate variability, providing invaluable information about the daily challenges faced by people with ASD and aiding in personalized intervention strategies. Furthermore, the convergence of wearable technology with IoT infrastructure has immense potential to enhance the connectivity and data-driven insights of ASD-specific wearables, as by seamlessly integrating with smart home environments, educational settings, and healthcare systems, IoT-enabled ASD wearables could facilitate remote monitoring, personalized interventions, and real-time feedback loops; for example, wearable devices could sync with smart home sensors to create soothing environments tailored to individual sensory preferences, or connect with educational platforms to provide personalized learning experiences based on real-time behavioral data. Finally, applying deep learning algorithms to the vast datasets collected by ASD-specific wearable devices could unlock new insights and predictive capabilities, as by leveraging deep learning models trained on multimodal data streams that include physiological signals, behavioral patterns, and environmental factors, these wearables could identify subtle correlations, predict behavioral episodes, and recommend personalized interventions in real-time, enabling continuous refinement and adaptation of wearable technologies based on individual responses and long-term outcomes, leading to increasingly effective and personalized support for people with ASD.

### 6.3. Limitations

This review highlights several important aspects of wearable technologies for ASD, but also presents some limitations that should be considered when interpreting the findings. First, the review does not include clinical trials or comparative studies that assess the direct impact of these technologies on the quality of life of individuals with ASD. Although this review provides valuable insights into commercially available wearables, the lack of real-world evaluations limits its ability to determine their practical efficacy. Future research should incorporate clinical assessments to better understand how these devices perform in everyday settings.

Second, while this review systematically gathered and analyzed a broad range of studies, a formal methodological quality assessment (e.g., GRADE or risk-of-bias tools) was not employed. Including a quality evaluation in future work would provide additional rigor and confidence in the results.

Third, the process of reducing over 17,000 studies to a manageable number for analysis posed challenges due to the variability in study designs and incomplete reporting. Although the PRISMA guidelines were followed, some relevant studies may have been excluded. Research teams attempting similar large-scale reviews could benefit from using clear protocols, inter-rate reliability checks, and automated tools to manage large datasets and address incomplete studies. Finally, the lack of clarity surrounding the FDA approval status of many devices limits the certainty about their readiness for clinical use. The absence of regulatory approval details could hinder the adoption of these devices in healthcare. Despite these limitations, this review offers a comprehensive overview of wearable technologies relevant to ASD, providing a solid foundation for future research and clinical application.

## 7. Conclusions

The findings of this study offer a significant advancement in the evaluation and application of commercial wearable technologies for ASD management. By providing a broad review spanning multiple domains, including executive function, psychomotor skills, and BES, this study contributes to a more holistic understanding of how wearable devices may affect individuals with ASD. Unlike other more limited research that focuses on isolated aspects, the broad scope of this study ensures a more complete picture of the potential benefits and limitations of wearable technologies in the treatment of ASD. Through this analysis, we highlight the existing challenges that must be addressed to fully realize the potential of wearable technologies. These challenges include the need for more rigorous clinical validation, improved user-centered design, and greater regulatory approval. While there is a consensus in the literature on the value of these technologies, this review has identified key gaps, such as the lack of large-scale, real-world validation studies and the limited FDA clearance of many devices, which restrict their broader adoption and effectiveness. By advancing research in this area, the field can not only contribute to more personalized and effective interventions but also expand the broader understanding of ASD, opening new avenues for treatment and support.

## Figures and Tables

**Table 1:** This table describes the domains, variables, and associated techniques used in the assessment of ASD.

Domain	Variables	Techniques
Executive function	Structural characteristics of the brain, electrical activity in the brain, neurotransmitters in the brain, neurostimulation, prosody	fMRI, DTI, MRS, EEG, tDCS, TMS.
Psychomotor	Body movement, muscle response, HRV, BVP, BP, RR	Accelerometers, motion tracking systems, ECG, PPG, EMG, SpO2.
BES	Gastrointestinal alterations, HRV, HR, EDA, electrodermal, touch sensing, olfaction, auditory, prosody, facial expression	Video capsule endoscopy (CE), bacterial biosensors, ECG, EMG, TDDS, VNS, HRI tactile sensors, neuroimaging, and eye tracking.

**Table 2:** Commercial wearable devices for autism focusing on the executive function domain.

Device Brand	Device Model	Main Features	Sensor Used	FDA Support Status/Year
InteraXon	Muse S (Gen 2) [56]	Seven EEG sensors on forehead and ears, PPG, respiratory and body movement sensors.	EEG, PPG, Gyroscope, Accelerometer, Pulse Oximetry	Not reported
Emotiv	EPOC X [57]	Has 14 channels with saline pads, and IMU motion sensor detects mental commands and facial muscle signals via 8 frontal sensors.	EEG, Gyroscope, Accelerometer	Not reported
NeuroSky	Mindwave Mobile 2 [58]	Two sensors measure brain signals for concentration, attention, or relaxation.	EEG	Not reported
X-trodes	X-trodes Smart Skin [59]	Adhesive skin patch monitors heart, muscle, and brain activity.	EEG	510(k) clearance/2024
g.tec	g.NAUTILUS PRO [60]	Portable EEG for medical use, with dry/wet electrodes, lightweight, adjustable sensitivity.	EEG	510(k) clearance/2017
BioSerenity	ICECap [61]	Disposable EEG grid in various sizes.	EEG	510(k) clearance/2021
Cumulus	Cumulus [62]	Sixteen electrodes for precise contact, fabric padding, water-resistant.	EEG	510(k) clearance/2023
Mendi	Mendi [63]	Forehead device measures brain activity with fNIRS sensors and detects blood flow.	fNIRS	Not applicable
FocusCalm	FocusCalm [64]	Real-time brain activity display via a FocusCalm score indicating stress levels	EEG	Not reported
Neurotechnology	BrainAccess [65]	Dry contact electrodes, hyperscanning for multi-person EEG and direct EEG data access.	EEG	Not reported
CGX	Quick-20 m [66]	Dry sensor device with 20 channels, shielding, and patented mechanisms for precise sensor positioning.	EEG	510(k) clearance/2021
OpenBCI	Ultracortex Mark IV [67]	A 3D-printable EEG device, compatible with OpenBCI. Records EEG, EMG, and ECG; 16 channels and dry sensors.	EEG, EMG, and ECG	Not reported
Zeto	Zeto EEG [68]	Nineteen dry electrodes and active electrode technology.	EEG	510(k) clearance/2018
X.on	X.on EEG [69]	One-size-fits-all design and 7 passive sponge electrodes.	EEG	Not reported
Macrotellect	BrainLink [70]	Three dry electrodes to measure brain waves, heart rate, and temperature.	EEG	Not reported
Neeuro	SenzeBand [71]	Captures EEG signals with 7 dry electrodes, interpreting them into mental states such as attention and relaxation. Machine learning algorithms provide biofeedback for real-time interventions in brain health problems.	EEG	Not reported
OMNIFIT Brain	OMNIFIT Brain [72]	It provides real-time biofeedback through machine learning, utilizing 7 dry electrodes.	EEG	Not reported
Advanced Brain Monitoring	B-Alert [73]	Automated wireless impedance checks, accelerometer, data quality monitoring, and 20 EEG channels with wet electrodes.	EEG, ECG, EOG, EMG,	510(k) clearance/2015
Wearable sensing	DSI-24 [74]	Provides motion immunity for real-world and VR/AR applications; 19 dry electrodes and 3 auxiliary sensors.	EEG	Not reported
Enobio	Enobio 32 [75]	A 32-channel EEG recording. High-density clinical electrodes or dry electrodes for out-of-laboratory recording.	EEG	510(k) clearance/2023
mbraintrain	Smarting PRO [76]	Artifact removal, up to 64 channels, 3D motion tracking, and configurable ExG recording with 8 bipolar electrodes.	EEG	Not reported
Meta	Meta Quest Pro [77]	It consists of high-resolution LCDs, VR/MR sensors, noise-canceling microphones, integrated speakers, Meta Quest Touch Pro controls, and SLAM tracking.	VR/MR	Not applicable
ViewPoint system	VPS 19 [78]	Infrared camera eye tracking, bi-directional communication, auto-calibration, 30 fps recording, wide field of view.	Infrared	Not applicable
Apple	Apple Vision Pro [79]	Aluminum/glass design, elastic strap, dual-chip (Apple M2/R1), micro-OLEDs (4K), eye/hand tracking, spatial audio.	Cameras	Not applicable
Tobii	Tobii Pro Glasses 3 [80]	Has 16 illuminators, 4 eye cameras, 106° scene camera, microphone, accelerometer, gyroscope, magnetometer, corneal reflex eye tracking, stereo geometry, and 50 Hz or 100 Hz sampling rate.	Cameras	Recall
EyeTribe	EyeTribe [81]	Compact eye-tracking device for desktops/tablets. Tracks eye movements via infrared technology.	Infrared	Not reported
X-trodes	XTR FEMG16 [82]	Patch with a 16-channel dry electrode array for monitoring facial EMG and EEG signals.	EMG, EEG	510(k) clearance/2024
PlatoScience Medical	PlatoWork headset [83]	Controlled by PlatoApp, offers six tDCS modes, three electrodes for precise positioning, exclusive to healthcare professionals.	tDCS	Not reported
LIFTiD	LIFTiD [84]	The device enhances concentration/alertness with low-level electrical currents.	tDCS	Not reported
Flow	Flow tDCS [85]	Depression treatment stimulates the brain’s frontal area, regulating mood, sleep, and motivation.	tDCS	FDA Breakthrough Device/2023
iMotions	iMotions Eye Tracking Glasses [86]	They capture real-world gaze data, offering over 30 metrics, areas of interest, live streaming, and automated quality assurance.	Camera	Not reported

**Table 3:** Summary of commercial wearable devices and sensor technologies for psychomotor assessment.

Device Brand	Device Model	Main Features	Sensor Used	FDA Support Status/Year
Ultraleap	Leap Motion Controller 2 [87]	Immersive hand tracking with high sensitivity, fast frame rates, and 27 joint details.	IR camera	Not applicable
BTS Bioengineering	Smart-Dx Evo [88]	A total of 256 infrared cameras, with a pixel resolution of 25 and a frame rate of 150.	IR camera	Not reported
BTS Bioengineering	G-walk [89]	Employs sensor fusion, which combines an accelerometer, gyroscope, and magnetometer for accurate multi-axis motion tracking.	Inertial Measurement Unit (IMU)	Not reported
BTS Bioengineering	P-6000 [90]	Features triaxial platforms, strain gauge sensors, and flexible positioning for biomechanical analyses.	Strain Gauge	Not reported
eSUN 3D	iSUN Gait Sensor Board [91]	Matrix pressure sensors with 3600 high-sensitivity sensors for analyzing static and dynamic foot data.	Pressure	Not reported
Elitac Wearables	SmartShirt [92]	Has 2 stretch and 4 movement sensors for motion analysis, central electronic processing, and 2 vibration motors for feedback.	Stretch and movement	Not reported
Hexoskin	Astroskin [93]	Real-time biometric monitoring for vital signs, including ECG, oxygen levels, respiration, and skin temperature.	ECG, SpO_2_, RR, and temperature	Not reported
Equivital	Eq lifemonitor [94]	Provides multi-parameter monitoring with minimal data loss and flexible sensor integration	ECG, temperature, and accelerometer	510(k) clearance/2012
Orpyx^®^	Orpyx Sensory Insole [95]	Monitors plantar pressure, temperature, step count, and adherence to reduce diabetic foot ulcer risk.	Pressure, temperature	510(k) clearance/2020
Myontec	Mbody [96]	The 8-channel EMG monitors forearm, biceps, deltoid, and trapezius muscles, along with IMU sensors for bending positions	EMG	Not reported
Delsys	Trigno Avanti [97]	Has 1 EMG, hasta 6 IMU	EMG, IMU	Not reported
BitBrain	Versatile Bio [98]	It records 21 to 35 channels of electrical and analog biosignals, movement, and behavior data.	ECG, galvanic skin response (GSR), EMG, IMU	Not reported
Spire Health Tag	Spire [99]	Tag that adheres to any cloth, and monitors stress levels, sleep, HR, and respiratory patterns.	Capnographer, ECG, and accelerometers.	Not applicable
HealthWatch Technologies	Master Caution [100]	Monitors cardiac ischemia, arrhythmias, respiration, and vital signs. Detects falls, inactivity, and skin temperature. Can be used both inside and outside hospital settings.	3–15 lead ECG monitoring; the garment is the sensor	510(k) clearance/2015
Nuubo	Nuubo Wearable ECG [101]	Features flexible, stretchable textile electrodes for high-quality ECG signals and patient comfort.	ECG	Not reported
Ambiotex	Ambiotex Smart Shirt [102]	Records pulse rate, breathing, heart rate variability, calorie consumption, and physical activity in real-time.	ECG and HR	Not reported
Sensoria	T-Shirt Short Sleeve + HRM [103]	Smart T-shirt that monitors heart rate, is compatible with various devices and apps, and is designed for comfort.	HR	Not reported
Oura [104], ringConn [105], ultrahuman [106], etc	Several models	-	Temperature, optical, infrared, SpO_2_, PPG	Not applicable
HEALBE [107], ViSi Mobile, Fitbit [108], etc.	Several models	-	Bioimpedance, IMU, temperature, SpO_2_, PPG,	Not applicable
Apple watch [109], Samsung [110], Huawei [111], etc.	Several models	-	IMU, PPG, SpO_2_, temperature, PPG	Not applicable
Sennheiser	Momentum Sport [112]	Headphones with adaptive noise cancelation, heart rate and temperature monitoring.	PPG and temperature	Not applicable
Bose	SoundSport Pulse [113]	Headphones with a heart rate sensor.	PPG	Not applicable
SBS	Heart Rate Runner [114]	Earphones with heart rate monitoring.	PPG	Not applicable
Philips	TAA4205BK [115]	Earphones with integrated heart rate monitoring.	PPG	Not applicable
MAFF Fitness	MAF HR+ Earbuds [116]	Earphones with clinical-grade heart rate monitoring	PPG	Not applicable
Komodo Technologies	AIO smart sleeve [117]	Compression sleeve with heart rate, ECG, sleep monitoring, and Bluetooth connectivity.	PPG	Not reported
Vivalink	Vivalink’s Wearable ECG Patch Monitor [118]	Reusable ECG patch providing continuous heart rate, ECG, and multiple health metric monitoring.	ECG	Not reported
BioIntellisense	Biobutton [119]	Medical-grade wearable for continuous monitoring of skin temperature, respiratory rate, and heart rate, with up to 16 days of battery life.	PPG, temperature, gait analysis	510(k) clearance/2022
Cosinuss	c-med° alpha [120]	A medical device that continuously streams core body temperature, heart rate, and blood oxygen saturation from a small, in-ear sensor.	PPG, temperature, SpO_2_	Not reported
Kenzen	Kenzen monitors [121]	Prevent heat-related injuries through real-time alerts, analytics, and predictive information to improve workplace safety.	PPG, temperature	Not reported
VitalConnect	Vital Patch RTM [122]	Features real-time data transmission, cloud integration, and analytics for detecting arrhythmias and other cardiac events.	ECG, RR, BP, SpO_2_	510(k) clearance/2020
Preventice	Bodyguardian Heart [123]	Provides a diagnostic and monitoring solution for cardiac patients.	Accelerometer, PPG	510(k) clearance/2012
Health Care Originals	ADAMM-RSM [124]	A device designed for continuous respiratory monitoring, especially for the treatment of chronic diseases such as asthma.	PPG and RR	Not reported
Biobeat	Biobeat Chest patch [125]	Continuously monitors multiple vital signs and performs real-time data analysis.	PPG	510(k) clearance/2019
Scosche	Rhythm24 [126]	Armband heart rate monitor.	ECG	Not reported
Philips	Philips extended Holter—ePatch [127]	Provides continuous, high-quality ECG data for 24 h for up to 14 days	ECG	Not reported
SmartCardia	SmartCardia 7L Patch [128]	Near real-time transmission of 7 ECG leads and vitals for 7/14 days.	ECG	510(k) clearance/2023
Wellue	Touchscreen ECG Monitor [129]	Hands-on ECG for quick checks and cable recording for noise-free signal. Duration options: 30 s, 60 s, 5 min.	ECG	Not reported
Wellue	Checkme [130]	Portable vital signs monitor that features real-time data display and data storage for long-term monitoring.	ECG and SpO_2_	510(k) clearance/2019
AliveCor	KardiaMobile [131]	Fingertip ECG detection.	ECG	510(k) clearance/2021
Emay	Portable EKG Moni [132] to	Different ways of recording: Hand-to-Hand, Hand-to-Chest, Hand-to-Leg	ECG	Not reported
ECG Check	Easy [133]	Records for 30 s.	ECG	Not reported
Heartcheck	Heartcheck Palm [134]	Only 30 s.	ECG	Not reported
Dimetek	Color ECG Recorder [135]	Supports skin touch or electrode connection, records ECG up to 32 h, and offers real-time monitoring with visual/audio alarms.	ECG	Not reported
Heal Force	Easy ECG Monitor—PC-80A [133]	Palm monitor.	ECG	Not reported
Quasar	PSM [136]	Records ECG, skin temperature, and 3-dimensional acceleration.	ECG, temperature, accelerometer	Not reported
iRhythm	Zio monitor [137]	Continuous cardiac monitor that can be worn for up to 14 days.	ECG	Not reported
Savvy	Savvy ECG [138]	Real-time on-screen ECG graph	ECG	Not reported
Philips	Biosensor BX100 [139]	Collect data up to 115 h and will attempt to transmit stored data for up to 120 h.	ECG	510(k) clear-ance/2020
BTS Bioengineering	Freeemg [140]	Surface electrodes.	EMG, electro goniometers and footswitch	Not reported
Senzime	TetraSensitive [141]	EMG sensor for sensitive skin, ultrasoft material.	EMG	Not reported
Elemyo	MYOstack v1.1 [142]	Simultaneous registration of 9 muscles with dry skin contact.	EMG	Not reported
MR EMG	MR EMG [143]	Simultaneous use of up to four sensors.	EMG	Not reported
Cometa	Picoblue [144]	Allows up to 4 sensors and 1000 Hz sampling.	EMG	Not reported
Noraxon	Ultium EMG [145]	Can activate IMU data or combine EMG with acceleration data.	EMG, IMU	Not reported
Shimmer	Shimmer3 EMG Unit [146]	Standard ECG functionality, two channels for EMG, and simultaneous measurement with 10DOF kinematic data.	EMG	Not reported
Smartex	Wearable Wellness System [147]	It includes textile sensors, activity classification, and energy expenditure, with comfort suitable.	ECG, accelerometer	Not reported

**Table 4:** Overview of commercial wearable devices and their features for BES management in ASD.

Device Brand	Device Model	Main Features	Sensor Used	FDA Support Status/Year
Medtronic	PillCam SB 3 [148]	Minimally invasive, ingestible capsule for small bowel visualization in clinic or hospital settings	Camera	510(k) clearance/2021
Check-cap	C-Scan [149]	Scans colon, communicates with C-Scan Track, and data are analyzed with C-Scan View after excretion.	Camera	Submission process/2021
G-Tech Medical	GutTracker [150]	Measures and analyzes gastrointestinal (GI) motility by capturing myoelectric signals from the digestive system	EMG	510(k) clearance
Biocam	Biocam [151]	Image analysis will be conducted by an artificial intelligence model	Camera	Not reported
IntorMedic	MiroCam Navi [152]	Controlled by a hand-held magnet, 3 FPS, 170° view, 9 h operation, 320 × 320 resolution with HDR	Camera	510(k) clearance/2018
Olympus	ENDOCAPSULE 10 [153]	Intuitive 3D tracking locates the capsule in the small intestine, wide 160° view	Camera	510(k) clearance/2014
CapsoVision	CapsoCam Plus [154]	Has 360° panoramic direct lateral view.	Camera	Recall
Jinshan	Omom HD [155]	Has 172° angle view, 0-50 mm depth of field, 4 LED lights	Camera	Not reported
Vagustim	Vagustim [156]	Non-invasive bilateral auricular	-	Not reported
Soterix Medical	RELIfit-Tragus [157]	Clips around the ear	-	Not reported
Touchpoint	Touchpoint [158]	Gentle, alternating vibrations	-	Not applicable
Apollo Neuro	Apollo [159]	Soothing vibrations		Not applicable
Sensate	Sensate 2 [160]	Soft vibrations and soothing sounds	-	Not applicable
Feelmore	Cove [161]	Mechanical stimulation behind each ear		Not applicable
Embr Wave	Embr Wave 2 [162]	A thermoelectric heat pump that generates cool or warm sensations	-	Not applicable
Sennheiser	Conversation Clear Plus [163]	Earbuds are designed to improve speech clarity in noisy environments	Microphone	Not reported
Neosensory	Neosensory Duo [164]	It uses vibrations to retrain the brain, helping to reduce the perception of tinnitus over time.	Microphone	Not reported
Nuheara	IQbuds2 MAX [165]	Smart hearing aids are designed to improve sound clarity in a variety of environments.	Microphone	Not reported
Olive Union	Olive Max [166]	Offers customizable sound amplification with Bluetooth connectivity for streaming and phone calls.	Microphone	510(k) clearance/2023
alango	BeHear NOW [167]	Offers features such as customizable sound amplification and music streaming	Microphone	Not reported
BitBrain	Ring [168]	Provides wireless data transmission.	GSR	Not reported
Empatica	Empatica Health Monitoring Platform [169]	Remote health monitoring solution that continuously tracks and analyzes physiological data; the platform utilizes the EmbracePlus wearable device.	GSR, PPG, IMU, Temperature	510(k) clearance/2022

## Data Availability

The data presented in this study are available on request from the corresponding author. The data are not publicly available due to privacy concerns of the users involved in the study.
